# Clinical Applications and Therapeutic Potential of Nano-Bio Fusion Gel in Oral Soft Tissue Therapy: A Critical Narrative Review

**DOI:** 10.3390/gels12050429

**Published:** 2026-05-14

**Authors:** Gábor Kammerhofer, Ákos Tamás Nagy, Árpád Joób-Fancsaly, György Szmirnov, Ilona Szmirnova, Dániel Végh, Márton Kivovics, György Szabó, Zsolt Németh

**Affiliations:** 1 Department of Oro-Maxillofacial Surgery and Stomatology, Semmelweis University, 1085 Budapest, Hungary; joob.fancsaly.arpad@semmelweis.hu (Á.J.-F.); szmirnov.gyorgy@semmelweis.hu (G.S.); szmirnova.ilona@semmelweis.hu (I.S.); szabo.gyorgy2@semmelweis.hu (G.S.); nemeth.zsolt@semmelweis.hu (Z.N.); 2 Faculty of Dentistry, Dental Student, Semmelweis University, 1088 Budapest, Hungary; nagy.akos02@stud.semmelweis.hu; 3 Department of Prosthodontics, Semmelweis University, 1088 Budapest, Hungary; vegh.daniel@semmelweis.hu; 4 Department of Community Dentistry, Semmelweis University, 1088 Budapest, Hungary; kivovics.marton@semmelweis.hu

**Keywords:** gingivitis, periodontitis, oral mucositis, oral wound, propolis, vitamin C, vitamin E, oral gel, bioadhesive gel, mucoadhesive gel, nano-bio fusion gingival gel

## Abstract

Oral mucosal and periodontal diseases are commonly associated with persistent inflammation, oxidative stress, impaired wound healing, and reduced oral health-related quality of life. Nano-Bio Fusion (NBF) gingival gel is a bioadhesive nano-formulated oral gel containing propolis, vitamin C, and vitamin E, developed for local application under oral soft tissue conditions. This critical narrative review aimed to evaluate the currently available evidence regarding the clinical applications, safety profile, and therapeutic potential of NBF gel in oral soft tissue therapy. A structured non-systematic literature search was performed using PubMed, Embase, Cochrane Library, and Google Scholar, and 16 relevant studies were included. The available evidence suggests that NBF gel may provide clinical benefits as an adjunct to non-surgical periodontal therapy, with reported improvements in plaque and gingival indices, periodontal probing depth, clinical attachment level, wound healing, and pain-related outcomes. In addition, potential beneficial effects have been reported in oral surgery-related wound healing, alveolar osteitis, desquamative gingivitis, erosive lichen planus, and xerostomia-associated mucositis. Several studies reported outcomes comparable to conventional therapies, including chlorhexidine-based regimens and locally delivered antimicrobials; however, the evidence remains heterogeneous and limited. Furthermore, the proposed biological mechanisms, including antioxidant, antimicrobial, and tissue-modulating effects, are not yet fully supported by mechanistic or pharmacokinetic evidence. The currently available literature is limited by heterogeneity in study design, small sample sizes, short follow-up periods, and limited independent validation. Therefore, further well-designed, adequately powered randomized controlled trials with standardized methodologies are required to better define the clinical role of NBF gel in evidence-based oral soft tissue therapy.

## 1. Introduction

Oral mucosal and periodontal diseases are among the most prevalent inflammatory conditions affecting the oral cavity and are associated with substantial impairment in oral health-related quality of life (OHRQoL) [1,2,3,4]. These conditions range from plaque-induced gingivitis and chronic periodontitis to immune-mediated mucosal disorders such as erosive oral lichen planus (OLP) and desquamative gingivitis. Their pathogenesis is characterized by a complex interaction between microbial dysbiosis, host inflammatory responses, oxidative stress, and impaired tissue repair mechanisms [5,6,7,8]. Persistent inflammation promotes the excessive production of reactive oxygen species (ROS), which contributes to connective tissue degradation, epithelial damage, and periodontal breakdown. In addition, oral surgical procedures may further compromise tissue healing. Delayed epithelialization, postoperative pain, and local inflammation are frequently observed following procedures such as free gingival graft (FGG) harvesting or tooth extraction complicated by alveolar osteitis (dry socket) [9,10]. Collectively, these conditions highlight the need for therapeutic approaches capable of modulating inflammation while supporting soft tissue healing and local tissue stability.

Conventional topical therapies used in oral medicine and periodontology include chlorhexidine gluconate (CHX), corticosteroids, and local antimicrobial delivery systems. Although CHX remains widely used for chemical plaque control, its long-term application is limited by adverse effects including tooth staining, taste alteration, and potential cytotoxic effects on oral fibroblasts [10,11,12]. Similarly, prolonged corticosteroid therapy may increase the risk of mucosal atrophy and opportunistic fungal infections, particularly in chronic immune-mediated mucosal diseases [2,13]. Local drug delivery (LDD) systems, including tetracycline-containing fibers and conventional polymer-based gels, have also been introduced to improve antimicrobial delivery within periodontal pockets. However, the oral environment presents considerable challenges for topical therapies due to continuous salivary flow, mechanical clearance, and limited retention time at the target site [14,15,16,17]. Consequently, there remains ongoing interest in bioadhesive and nanotechnology-based delivery systems that may improve local retention and therapeutic efficacy while minimizing adverse effects.

NBF technology represents a nanotechnology-based bioadhesive oral delivery approach designed for the local administration of antioxidant and biologically active compounds [2,15,16,17]. The NBF gingival gel contains propolis extract, sodium ascorbyl phosphate (vitamin C), and tocopherol acetate (vitamin E) incorporated into a nano-formulated bioadhesive matrix. Propolis is a natural resinous substance produced by honeybees and contains numerous biologically active compounds, including flavonoids and polyphenols, which have demonstrated antimicrobial, antioxidant, and anti-inflammatory properties in experimental and clinical studies [18,19,20,21,22,23]. Vitamin C is involved in collagen synthesis and fibroblast function, while vitamin E contributes to the stabilization of cellular lipid membranes and may reduce oxidative stress-associated tissue damage [2,14,24,25,26]. The nano-formulated architecture and bioadhesive properties of the gel may facilitate prolonged surface contact and local retention of active compounds within the oral cavity, potentially supporting their therapeutic activity in environments characterized by continuous salivary flow [16,17].

In recent years, several clinical studies have investigated the application of NBF gel in various oral soft tissue conditions, including gingivitis, chronic periodontitis, alveolar osteitis, oral mucositis, desquamative gingivitis, and wound healing following oral surgical procedures [1,2,8,9,10,14,15,16,17]. Reported clinical outcomes include reductions in inflammatory indices, improvements in wound healing, and decreased postoperative discomfort. However, the currently available evidence remains heterogeneous with respect to study design, treatment protocols, and outcome assessment, while mechanistic and formulation-related data remain limited. Therefore, a critical synthesis of the available literature may help to better define the current evidence base and the potential clinical role of NBF gel in oral soft tissue therapy.

The present study aimed to critically review the currently available evidence regarding the clinical applications, safety profile, and therapeutic potential of NBF gel in the management of oral soft tissue conditions. By synthesizing the available evidence, this review aims to better define the potential clinical role of NBF gel as an adjunctive therapeutic approach in contemporary oral soft tissue therapy [27,28,29,30,31,32,33,34].

To provide a critical overview of the currently available evidence, a structured non-systematic literature search was conducted using PubMed, Embase, Cochrane Library, and Google Scholar. The search included studies published up to March 2026 and was based on combinations of keywords including “gingivitis”, “periodontitis”, “oral mucositis”, “wound healing”, “propolis”, “vitamin C”, “vitamin E”, “bioadhesive gel”, “mucoadhesive gel”, and “Nano-Bio Fusion gingival gel”, combined using Boolean operators (AND/OR). The review focused on studies investigating the clinical applications of NBF gingival gel and related propolis-based bioadhesive oral formulations under oral soft tissue conditions. Titles, abstracts, and subsequently full texts were screened for relevance. Studies were considered eligible if they evaluated clinical or therapeutically relevant applications of NBF gel in oral soft tissue therapy and reported outcomes related to inflammation control, wound healing, or symptom improvement. Due to the heterogeneity of study designs, clinical indications, and outcome measures, the findings were synthesized narratively rather than through quantitative meta-analysis. Relevant studies were screened and qualitatively analyzed with regard to clinical indications, treatment protocols, and reported therapeutic outcomes associated with the use of NBF gingival gel and related bioadhesive oral formulations in oral soft tissue therapy. A total of 16 studies were considered relevant and included in the present review. The present critical narrative review aimed to evaluate the currently available evidence regarding the clinical efficacy, safety profile, and therapeutic potential of NBF gel in oral soft tissue therapy.

## 2. Results and Discussion

This review of the clinical literature identified 16 primary studies investigating the therapeutic application of NBF gingival gel across various oral pathologies (Table 1, Table 2 and Table 3). The included studies were conducted across diverse geographical regions, including Egypt, Hungary, India, Saudi Arabia, Spain, North Macedonia, and South Korea, reflecting the current international distribution of the available clinical evidence regarding NBF gel applications. These studies encompass a broad spectrum of clinical conditions, including chronic periodontitis, gingivitis (including orthodontic-induced cases), oral surgery-related complications (such as alveolar osteitis and palatal wounds), and complex oral medicine conditions, including desquamative gingivitis, erosive lichen planus, and xerostomia-associated mucositis.

### 2.1. Clinical Outcomes in Periodontology and Gingival Health

The primary application of NBF gel in the reviewed literature was as an adjunct to non-surgical periodontal therapy (NSPT), particularly scaling and root planing (SRP). In the randomized controlled trial (RCT) conducted by Aeran et al. [32], the efficacy of NBF gel was compared with 0.2% CHX gel in 45 patients. The NBF group demonstrated a reduction in periodontal probing depth (PPD) from 5.62 ± 0.44 mm at baseline to 3.12 ± 0.32 mm at 3 months, with significantly greater reductions compared to the CHX group (*p* < 0.05). Similarly, Srivastava et al. [33] reported in a split-mouth study (*n* = 20) that NBF-treated sites exhibited significantly lower plaque index (PI) (*p* = 0.003) and gingival index (GI) (*p* = 0.001) values compared to SRP alone at 3 months. In addition, significantly greater gains in clinical attachment level (CAL) were observed in the NBF group (*p* = 0.005), although the long-term clinical relevance of these findings remains to be further clarified. Dayakar et al. [31] compared NBF gel with Blue M gel, an oxygen-releasing formulation, in patients with chronic periodontitis. Although both treatments demonstrated clinical improvements, the NBF gel group showed greater reductions in GI (0.82 ± 0.12) compared to the Blue M group (1.04 ± 0.15). However, the mechanistic basis of these differences remains insufficiently characterized.

### 2.2. Local Drug Delivery (LDD) and Microbiological Effects

A key question in periodontology is whether bioadhesive gels may serve as adjunctive alternatives to traditional local antibiotic therapies. Bodduru et al. [14] compared NBF gel with tetracycline-impregnated fibers and reported comparable reductions in PPD (~2.1 mm at 3 months, *p* > 0.05), suggesting that NBF gel may represent a potential non-antibiotic adjunctive approach in periodontal therapy. This observation may be clinically relevant in the context of increasing concerns regarding antimicrobial resistance. Supporting microbiological findings were reported by Debnath et al. [17], who observed reductions in anaerobic periodontal bacterial counts, measured as colony-forming units, in NBF-treated sites compared to control sites.

### 2.3. Gingivitis Management in Orthodontic Patients

In less severe inflammatory conditions, clinical improvements associated with NBF gel application have also been reported. Singh et al. [34] observed reductions in both GI and papillary bleeding index (PBI) after 1 month of treatment. In patients undergoing fixed orthodontic treatment, Alam and Ganji [30] reported that twice-daily application of NBF gel reduced the PI from 1.82 ± 0.22 to 0.64 ± 0.14 over a 90-day period. These findings may be clinically relevant, as orthodontic patients often experience difficulties in maintaining effective mechanical plaque control during treatment.

### 2.4. Clinical Evidence in Oral Surgery and Wound Healing

Wound healing and postoperative symptom control represent clinical areas in which several studies have reported potentially beneficial effects associated with NBF gel application. Alveolar osteitis is characterized by intense pain and delayed socket healing. Khan et al. [9] compared NBF gel with organic olive oil in 90 patients and reported reductions in pain scores during the postoperative period. In the NBF group, the mean visual analog scale (VAS) score decreased from 8.4 ± 0.8 at baseline to 1.2 ± 0.4 by day 7, with significantly lower pain scores compared to the control group (*p* < 0.001). González-Serrano et al. [15] reported that post-extraction application of NBF gel was associated with reduced postoperative pain and a lower incidence of dry socket in their study population. Healing of the palatal donor site following FGG harvesting is typically associated with considerable postoperative discomfort. Abdelrehim et al. [10] demonstrated that the combined use of NBF gel and a palatal stent resulted in significantly higher healing index values at day 3 (*p* = 0.017). In addition, patient satisfaction scores were higher in the NBF group (8.8 ± 0.9) compared to the control group (6.4 ± 1.2), suggesting a potential beneficial effect on postoperative comfort. Fidoski et al. [26] evaluated 90 patients with various oral wounds and reported improved healing outcomes according to the Landry index in the NBF-treated group compared to placebo controls. These findings are consistent with early clinical observations reported by Chae et al. [3], who described ulcer healing following NBF gel application without serious adverse effects.

In oral medicine applications, several studies have also reported improvements in mucosal conditions associated with chronic inflammation and xerostomia. In the largest study included in this review (*n* = 127), Szabó et al. [1] reported improvements in mucosal status and quality of life (QoL) parameters in patients with xerostomia following NBF gel application. Among patients receiving combined treatment with NBF gel and pilocarpine, the World Health Organization (WHO) mucositis scale improved by an average of 1.8 points. González-Serrano et al. [2] reported a reduction in the desquamative gingivitis clinical score (DGCS) from 6.4 ± 1.8 to 2.2 ± 1.1 after 4 weeks of treatment (*p* < 0.001). In a case report, Popovska et al. [14] described symptomatic improvement and lesion resolution in a patient with erosive lichen planus following repeated NBF gel application.

### 2.5. Mechanisms of NBF Technology

The clinical effects associated with NBF gel application may partly be related to its bioadhesive delivery characteristics and the biological activity of its active components. The oral cavity represents a challenging environment for topical drug delivery because continuous salivary flow and mechanical clearance may reduce local retention of conventional formulations. NBF technology utilizes a nano-formulated bioadhesive matrix containing propolis, vitamin C, and vitamin E. The small particle size and bioadhesive properties of the formulation may contribute to prolonged surface contact and improved local retention of active compounds within oral soft tissues [15,16,17]. However, important formulation-related parameters, including tissue penetration, local bioavailability, and release kinetics, remain insufficiently characterized in the currently available literature.

Inflammation in periodontal and mucosal diseases is closely associated with oxidative stress and excessive production of reactive oxygen species (ROS), which may contribute to tissue damage and delayed healing [5,6,7,8]. Vitamin C functions as an antioxidant and an essential cofactor in collagen synthesis, while vitamin E may contribute to stabilization of cellular lipid membranes and reduction of oxidative stress-associated tissue injury [2,14,24,25,26]. Propolis contains flavonoids and polyphenolic compounds that have demonstrated antimicrobial and anti-inflammatory properties in experimental studies [18,19,20,21,22,23]. Collectively, these components may contribute to modulation of the local inflammatory environment and support soft tissue healing processes.

Several studies included in this review reported reductions in pain scores and improvements in wound healing outcomes following NBF gel application [9,10,15,26]. These effects may partly be related to the protective bioadhesive properties of the hydrogel matrix, which may help maintain prolonged contact with exposed tissues. In addition, the currently available studies suggest that NBF gel is generally well tolerated, with no serious adverse effects reported. The absence of commonly reported side effects associated with certain conventional topical therapies, such as tooth staining, taste alteration, or mucosal irritation, may potentially support patient compliance during prolonged use.

**Figure 1:**
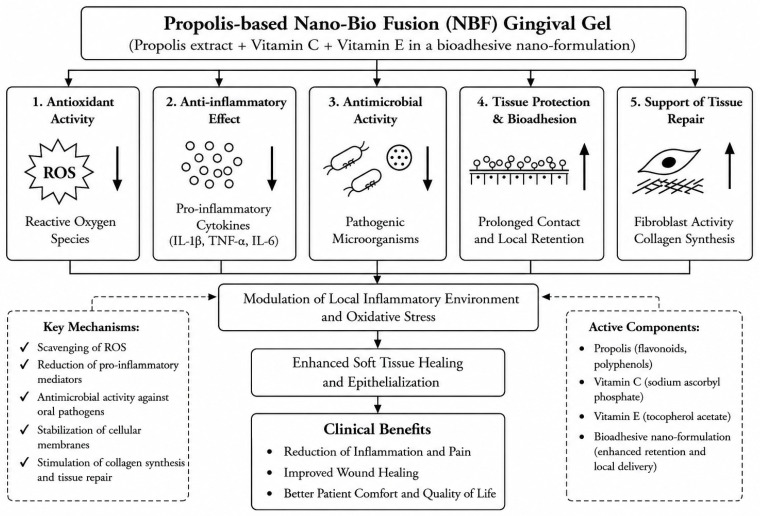
Proposed mechanisms of action and local delivery characteristics of propolis-based NBF gingival gel in oral soft tissue therapy, based on the available literature [35,36,37], created by the authors. Downward arrows (↓) indicate reduction or inhibition of reactive oxygen species, pro-inflammatory cytokines, and pathogenic microorganisms, whereas upward arrows (↑) indicate enhancement of local retention, fibroblast activity, and collagen synthesis.

The proposed mechanisms of action are summarized in Figure 1 [35,36,37].

### 2.6. Local Delivery Characteristics and Clinical Applicability

In addition to its biochemical properties, the clinical utility of NBF gingival gel is closely related to its local delivery characteristics. As a bioadhesive formulation, the gel may maintain prolonged contact with the oral mucosa, potentially enhancing the local availability of active compounds at the target site. This feature is particularly relevant in the oral environment, where continuous salivary flow and mechanical clearance can rapidly reduce the effectiveness of conventional topical agents. The semi-solid consistency of the formulation may facilitate retention within periodontal pockets and on mucosal surfaces, supporting both professional and at-home application. Furthermore, the combination of mechanical coverage and sustained local presence may contribute to the protection of exposed tissues and stabilization of the local microenvironment, thereby facilitating conditions favorable for wound healing and tissue regeneration [2,3,8,15,26].

According to manufacturer-provided formulation information, NBF gingival gel also contains supportive excipients and compounds, including cellulose gum, PEG-1500, glycerin, xylitol, menthol, and *Mentha piperita* oil, which may contribute to its bioadhesive behavior, local retention capacity, and patient tolerability. In particular, viscosity-enhancing components and the hydrogel-like matrix may support prolonged mucosal contact and improve patient comfort during application. The inclusion of menthol and peppermint oil may additionally provide local soothing and sensory effects; however, their specific contribution to the observed clinical outcomes remains unclear.

Despite these proposed formulation-related advantages, detailed physicochemical characterization of the gel remains limited in the currently available peer-reviewed literature. Parameters such as particle size distribution, polydispersity index, zeta potential, release kinetics, mucoadhesive strength, and long-term formulation stability have not yet been comprehensively investigated. Therefore, although the currently available clinical findings are encouraging, the mechanistic and pharmaceutical basis of the formulation requires further experimental validation.

### 2.7. Limitations of the Current Evidence

The currently available evidence on NBF gel remains limited by substantial heterogeneity in study design, clinical indications, treatment protocols, follow-up periods, and outcome measures. Although several randomized controlled trials have reported favorable clinical outcomes, many studies are characterized by small sample sizes, short follow-up durations, split-mouth designs, or lower levels of evidence. In addition, independent validation remains limited, and the predominance of positive findings may indicate potential publication bias. Important formulation-related parameters, including stability, release kinetics, tissue penetration, and local bioavailability, are also insufficiently characterized. In addition, transient yellowish-brown tooth discoloration and local sensory irritation, potentially related to propolis pigments and menthol-containing components, may occur in some patients and could influence patient acceptance during prolonged use. Furthermore, this article was designed as a critical narrative review rather than a systematic review; therefore, selection bias and incomplete literature capture cannot be entirely excluded. Further well-designed, adequately powered randomized controlled trials with standardized methodologies and long-term follow-up are required to better define the clinical role of NBF gel in oral soft tissue therapy.

### 2.8. Future Perspectives

The potential application of NBF gel in oral and maxillofacial conditions represents an area of growing clinical interest, particularly in relation to wound healing and local inflammation control. Based on the currently available evidence, potential future indications may include alveolar osteitis, peri-implant mucositis, peri-implantitis, oral mucositis, post-surgical soft tissue management, orthodontic gingivitis, and selected immune-mediated mucosal diseases. Particular attention may also be directed toward compromised healing conditions, including diabetes mellitus and medication-related osteonecrosis of the jaw (MRONJ), in which impaired wound healing and persistent inflammation play a central pathogenic role [38,39,40,41,42,43,44,45,46]. However, the currently available evidence for these applications remains limited, and most proposed indications have not yet been validated in adequately powered clinical trials. Therefore, future well-designed prospective and controlled studies are required to clarify the clinical efficacy, safety profile, and optimal therapeutic protocols of NBF gel in these settings.

## 3. Conclusions

Based on the currently available evidence, NBF gel may represent a promising adjunctive therapeutic approach in the management of various oral soft tissue conditions, including gingivitis, chronic periodontitis, alveolar osteitis, and mucosal lesions. Across predominantly randomized controlled trials, the application of NBF gel was associated with improvements in clinical parameters, including reductions in plaque and gingival indices, probing depth, clinical attachment loss, as well as enhanced wound healing and pain relief. In several studies, these outcomes were reported to be comparable to conventional therapies, including CHX-based regimens and locally delivered antimicrobials; however, the available evidence remains limited and heterogeneous. The included studies differ substantially in study design, intervention protocols, outcome measures, and follow-up periods, while many investigations were conducted with relatively small sample sizes or split-mouth designs. Consequently, the overall strength and generalizability of the current evidence remain limited. In addition, standardized treatment protocols and robust long-term clinical data are still lacking. Although no serious adverse effects were reported in the included studies, independent validation remains limited, and the predominance of positive findings may indicate potential publication or selection bias. Furthermore, despite the proposed biological mechanisms, the actual bioavailability, tissue penetration, and effective local concentrations of NBF gel components remain insufficiently characterized. Therefore, the proposed antioxidant, antimicrobial, and regenerative effects should be interpreted cautiously until further mechanistic and pharmacokinetic evidence becomes available. Future well-designed, adequately powered, independently conducted randomized controlled trials with standardized methodologies and extended follow-up are required to more clearly define the clinical role of NBF gel in evidence-based oral soft tissue therapy.

## Figures and Tables

**Table 1:** Overview of included studies according to geographical location, study design, and investigated clinical conditions.

Literature (Author, Year)	Country/Location	Type of Study	Clinical Condition
Abdelrehim et al. (2025) [10]	Cairo, Egypt	RCT (Parallel)	Palatal Wound, FGG
Szabó et al. (2024) [1]	Budapest, Hungary	Retrospective Study	Mucositis & Xerostomia
Dayakar et al. (2023) [31]	Karnataka, India	RCT (Split-mouth)	Chronic Periodontitis
Aeran et al. (2023) [32]	Uttarakhand, India	RCT (Comparative)	Chronic Periodontitis
Khan et al. (2023) [9]	Sakaka, Saudi Arabia	RCT (Double-blind)	Alveolar Osteitis (Dry Socket)
González-Serrano et al. (2023) [2]	Madrid, Spain	RCT (Double-blind)	Desquamative Gingivitis
Bodduru et al. (2022) [14]	Telangana, India	Comparative Study	Chronic Periodontitis
González-Serrano et al. (2021) [15]	Madrid, Spain	RCT (Split-mouth)	Alveolar Osteitis Prevention
Singh et al. (2020) [34]	India (Institutional)	RCT (Parallel)	Gingivitis
Vivek Srivastava et al. (2019) [33]	Aligarh, India	RCT (Split-mouth)	Chronic Periodontitis
Fidoski et al. (2017) [26]	Skopje, North Macedonia	RCT (Double-blind)	Oral Mucosa Wounds
Alam & Ganji (2016) [29]	Sakaka, Saudi Arabia	Prospective Study	Ortho-induced Gingivitis
Debnath et al. (2016) [17]	Bangalore, India	RCT	Chronic Periodontitis
Popovska et al. (2016) [13]	Skopje, Macedonia	Case Report	Erosive Lichen Planus
Chatterjee & Sneha (2014) [4]	Bangalore, India	Clinical Study	Gingivitis
Chae et al. (2007) [3]	Seoul, South Korea	Case Series	Various Mucosal Wounds

**Table 2:** Overview of interventions and study populations in the included studies.

Literature (Author, Year)	Research Area/Materials	Population (N)
Abdelrehim et al. (2025) [10]	NBF Gel + Palatal stent vs. Stent alone	26 (16 F, 10 M)
Szabó et al. (2024) [1]	NBF Gel vs. NBF Gel + Pilocarpine	127 (102 F, 25 M)
Dayakar et al. (2023) [31]	SRP + NBF Gel vs. SRP + Blue M Gel	32 (15 F, 17 M)
Aeran et al. (2023) [32]	SRP + NBF Gel vs. SRP + CHX Gel vs. SRP alone	45 (21 F, 24 M)
Khan et al. (2023) [9]	NBF Gel vs. Organic Olive Oil	90 (68 F, 32 M)
González-Serrano et al. (2023) [2]	NBF Gel vs. Placebo gel	22 (all F)
Bodduru et al. (2022) [14]	NBF Gel vs. Tetracycline fibers, LDD	20 (40 sites)
González-Serrano et al. (2021) [15]	NBF Gel vs. Placebo gel	13 (10 F, 3 M)
Singh et al. (2020) [34]	SRP + NBF Gel vs. NBF alone vs. SRP alone	7 (21 quadrants) (4 F, 3 M)
Vivek Srivastava et al. (2019) [33]	SRP + NBF Gel vs. SRP alone	20
Fidoski et al. (2017) [26]	NBF Gel vs. Placebo	90 (47 F, 43 M)
Alam & Ganji (2016) [29]	NBF Gel application (twice daily)	64
Debnath et al. (2016) [17]	SRP + NBF Gel vs. SRP alone	6 (76 sites)
Popovska et al. (2016) [13]	NBF Gel (3–5× daily)	1 (M)
Chatterjee & Sneha (2014) [4]	SRP + NBF Gel vs. SRP alone	15
Chae et al. (2007) [3]	NBF Gel application	5

**Table 3:** Overview of the main clinical outcomes associated with NBF gingival gel.

Literature (Author, Year)	Main Results
Abdelrehim et al. (2025) [10]	Significant improvement in healing index at day 3; higher patient satisfaction and lower pain scores.
Szabó et al. (2024) [1]	Long-term improvements in mucosal status and quality of life were reported.
Dayakar et al. (2023) [31]	Both gels were effective, but NBF gel showed greater reductions in GI and PI.
Aeran et al. (2023) [32]	NBF gel group showed significantly higher reduction in PPD and CAL compared to CHX and SRP alone groups.
Khan et al. (2023) [9]	NBF gel provided significantly faster pain relief and socket healing starting from the third day.
González-Serrano et al. (2023) [2]	Significant reduction in DGCS and improvement in OHIP-14.
Bodduru et al. (2022) [14]	NBF gel efficacy was found comparable to tetracycline fibers in reducing PPD.
González-Serrano et al. (2021) [15]	Statistically significant reduction in postoperative pain during the first week after third molar surgery.
Singh et al. (2020) [34]	Adjunctive NBF gel application resulted in the highest reduction of gingival and papillary bleeding.
Vivek Srivastava et al. (2019) [33]	From baseline to 3 months, a statistically significant difference was observed between the groups in PI, GI, PPD, and CAL.
Fidoski et al. (2017) [26]	NBF nano-emulsion complex significantly promoted soft tissue healing and epithelization.
Alam & Ganji (2016) [29]	Significant reduction in PI, PBI, and PPD over a 90-day observation period.
Debnath et al. (2016) [17]	Significant improvement in CAL and PPD; reduction in anaerobic microbial counts.
Popovska et al. (2016) [13]	Rapid healing of erosive lesions and complete symptomatic relief within a short period.
Chatterjee & Sneha (2014) [4]	NBF gel was an effective herbal alternative to CHX in reducing inflammation.
Chae et al. (2007) [3]	First clinical observation showing rapid healing of ulcers and inflammatory lesions without side effects.

## Data Availability

No new data were created or analyzed in this study. Data sharing is not applicable to this article.

## Abbreviations

**Abbreviation**	**Definition**
CHX	Chlorhexidine
CAL	Clinical attachment level
DGCS	Desquamative gingivitis clinical score
F	Female
FGG	Free gingival graft
GI	Gingival index (Löe and Silness)
IL-1β	Interleukin-1 beta
IL-6	Interleukin-6
LDD	Local drug delivery
M	Male
MRONJ	Medication-related osteonecrosis of the jaw
NBF	Nano-bio fusion (nano-emulsion formulation containing vitamin C, vitamin E, and propolis)
NSPT	Non-surgical periodontal therapy
OHIP-14	Oral health impact profile-14
OHRQoL	Oral health-related quality of life
OLP	Oral lichen planus
PBI	Papillary bleeding index
PPD	Periodontal probing depth
PI	Plaque index
PEG-1500	Polyethylene glycol 1500
QoL	Quality of life
RCT	Randomized controlled trial
ROS	Reactive oxygen species
SRP	Scaling and root planing
TNF-α	Tumor necrosis factor-alpha
WHO	World health organization
